# Hepatitis B e Antigen Induces NKG2A^+^ Natural Killer Cell Dysfunction via Regulatory T Cell-Derived Interleukin 10 in Chronic Hepatitis B Virus Infection

**DOI:** 10.3389/fcell.2020.00421

**Published:** 2020-06-03

**Authors:** Qingqing Ma, Xiaoyu Dong, Siyu Liu, Tao Zhong, Dandan Sun, Lu Zong, Changcheng Zhao, Qiong Lu, Min Zhang, Yufeng Gao, Ying Ye, Jun Cheng, Yuanhong Xu, Meijuan Zheng

**Affiliations:** ^1^Department of Clinical Laboratory, The First Affiliated Hospital of Anhui Medical University, Hefei, China; ^2^Department of Clinical Laboratory, Chaohu Hospital of Anhui Medical University, Chaohu, China; ^3^Department of Blood Transfusion, The First Affiliated Hospital of Anhui Medical University, Hefei, China; ^4^Department of Life Sciences and Medicine, The First Affiliated Hospital, University of Science and Technology of China, Hefei, China; ^5^Department of Infectious Diseases, The First Affiliated Hospital, Anhui Medical University, Hefei, China

**Keywords:** hepatitis B e antigen, HBV, NK cell, NKG2A, IL-10

## Abstract

Although persistent hepatitis B virus (HBV) infection is associated with natural killer (NK) cell dysfunction, it remains obscure whether HBV viral antigens are responsible for NK cell dysfunction in patients with chronic hepatitis B (CHB) infection. In this study, we found that the percentage of NK cells expressing the inhibitory receptor, NKG2A, was increased in CHB patients, and NKG2A blockade restored NK cell function. Furthermore, in CHB patients, the frequency of NK cells expressing NKG2A positively correlated with the number of regulatory T cells (Tregs) and production of interleukin-10 (IL-10) in these Tregs. Moreover, exposure of peripheral blood mononuclear cells (PBMCs) isolated from healthy controls to sera from CHB patients resulted in increased proportion of NKG2A^+^ NK cells; IL-10 blockade reduced the frequency of NKG2A^+^ NK cells while increasing the percentage of IFN-γ^+^ NK cells. In addition, stimulation of NK cells and Tregs from healthy controls with CHB sera together with anti-IL-10 antibody increased IFN-γ production in the culture supernatant. The frequencies of NKG2A^+^ NK cells and IL-10^+^ Tregs, along with serum levels of alanine transferase and HBV DNA, were significantly increased in CHB patients positive for the Hepatitis B e antigen (HBeAg, a marker of viral replication) when compared to HBeAg-negative CHB patients. Importantly, exposure of PBMCs from healthy controls to HBeAg resulted in increased IL-10 production but reduced levels of TNF and IFN-γ, and IL-10 blockade rescued the generation of TNF and IFN-γ in this assay. The reduced production of TNF and IFN-γ was also observed in NK cells and Tregs from healthy controls that were stimulated with HBeAg, while IL-10 blockade increased the secretion of these two cytokines. We conclude that HBeAg induces IL-10 production in Tregs, thereby leading to increased expression of NKG2A on NK cells, which contributes to NK cell dysfunction during CHB infection. These data suggest that HBeAg is associated with NK cell dysfunction in CHB.

## Introduction

Hepatitis B virus (HBV) infection is a major public health problem worldwide and individuals with chronic HBV (CHB) infection are at high-risk for the development of cirrhosis and hepatocellular carcinoma (HCC) (Lozano et al., 2012; Maini and Peppa, 2013; Tian et al., 2013). CHB is associated with ineffective antiviral immune responses (Dienstag, 2008; Li et al., 2015), and accumulating evidence supports a relationship between CHB infection and impaired natural killer (NK) cell cytotoxicity and cytokine secretion (Martinet et al., 2012; Zheng et al., 2018). Despite this association, the mechanisms involved in NK cell dysfunction in CHB patients are yet to be clarified.

NK cells are the predominant lymphocyte subpopulation in the liver, constituting ∼31% of intrahepatic lymphocytes (Racanelli and Rehermann, 2006; Peng et al., 2016). NK cell activity is regulated by the combination of activating and inhibitory receptors they express (Long et al., 2013; Zheng et al., 2018; El-Deeb et al., 2019). The chronic viral infection is associated with increased expression of inhibitory receptors on NK cells, which correlates with a poor decline in viral titers after therapy (Golden-Mason et al., 2011; Rehermann, 2013). Recently, NKG2A has been reported as a marker of NK exhaustion in the hepatitis C virus infection and it contributes to viral persistence (Zhang et al., 2019). During HBV infection, the expression of NKG2A on NK cells is elevated in patients with active CHB, and blocking NKG2A signaling increases NK cell cytotoxicity *in vitro* (Li et al., 2013). Furthermore, high levels of NKG2A expression on NK cells leads to NK cell exhaustion and is associated with poor prognosis for patients with HCC (Sun et al., 2017). Anti-NKG2A treatment has been suggested to enhance NK cell activity in cancer vaccinations (Haanen and Cerundolo, 2018).

Increased regulatory T cells (Tregs) and interleukin 10 (IL-10) levels in the circulation are associated with weak T cell responses in patients with CHB (Park et al., 2016). Tregs can inhibit NK and CD8^+^ T cell antiviral capacity through their secretion of IL-10 (Trehanpati and Vyas, 2017). Furthermore, high levels of IL-10 in patients with CHB inhibit IFN-γ production in NK cells (Peppa et al., 2010), and intrahepatic IL-10 contributes to the hyporesponsive state of NKG2A^+^Ly49^–^ NK cells in the liver (Lassen et al., 2010). Li et al. also found that hepatic Tregs contribute to NKG2A expression on murine NK cells, suggesting that reagents designed to block NKG2A signaling have considerable potential for application in the treatment of CHB infection (Li et al., 2013). Moreover, Hepatitis B e antigen (HBeAg, a marker of viral replication) has an important role in viral persistence, and is associated with dysfunctional T cell responses in patients with CHB infection (Tian et al., 2016; Yang et al., 2019), however, it is not clear whether viral factors are involved in the dysfunction of NKG2A^+^ NK cells in patients with CHB.

In this study, we found that increased percentages of NKG2A^+^ NK cells in peripheral blood correlated with HBV-DNA titers and that blocking NKG2A could restore the function of NK cells isolated from patients with CHB *in vitro*. We also observed a positive correlation between NKG2A^+^ NK cells and IL-10^+^ Tregs in patients with CHB. Moreover, exposure of peripheral blood mononuclear cells (PBMCs) or purified NK cells isolated from healthy controls to CHB sera resulted in impaired NK cell function. Meanwhile, HBeAg-positive patients had higher frequencies of NKG2A^+^ NK cells and Tregs than those who were HBeAg-negative. Our data demonstrate that HBeAg can induce IL-10 secretion in Tregs and that blocking IL-10 enhances NK cell function. Overall, our findings delineate a possible mechanism underlying the dysfunction of NKG2A^+^ NK cells in HBeAg-positive CHB patients and suggest that the HBeAg-IL-10-NKG2A^+^ NK cell axis is a potential therapeutic target in CHB patients.

## Materials and Methods

### Patients and Healthy Controls

The results reported in this study were generated from 69 patients with active CHB who had not received antiviral therapy, and 37 age- and sex-matched healthy controls (HCs). In addition, 15 patients with CHB post-therapy were recruited, and this group of patients were treated with entecavir (0.5 mg per day) for 6 months. All patients with CHB were characterized by serum alanine transferase (ALT) levels > 61 U/L and HBV-DNA levels > 2000 U/L, and none had overlapping infections with other hepatitis viruses, drug-induced hepatitis, alcoholic hepatitis, tumors, or autoimmune liver diseases. PBMCs were isolated from fresh blood using human peripheral blood lymphocyte isolation fluid (TBD Science, #LTS1077). The characteristics of enrolled patients with CHB and healthy controls, based on whole blood staining, are summarized in Table 1. All patients were diagnosed with CHB and all healthy donors were free from viral hepatitis, autoimmune hepatitis, and tumors. This study was approved by the local ethics committee of The First Affiliated Hospital of Anhui Medical University and the local ethics committee of Chaohu Hospital of Anhui Medical University.

**TABLE 1:** Clinical characteristics of enrolled subjects.

Clinical characteristics	CHB-actives	Healthy controls
Case	69	37
Sex (male)	50 (62.5%)	22 (59.5%)
Age, year [mean ± SEM]	39.4 ± 1.4	40.7 ± 1.9
ALT, U/L [mean ± SEM]	363.4 ± 56.5	23.2 ± 2.1
HBV DNA, U/m l [mean ± SEM]	(5.27 ± 1.02) E + 7	n.a.
HBsAg positive	69/69	n.a.
HBeAg positive	40/69	n.a.

*SEM, standard error of mean; n.a., not applicable.*

### Purification of Cells

NK cells were purified using a human NK cell Isolation Kit (Miltenyi Biotec, 130-092-957) and CD4^+^CD25^+^ Tregs were purified using a human CD4^+^CD25^+^ Regulatory T Cell Isolation Kit (Miltenyi Biotec, 130-091-301). Cell purity was determined by flow cytometry and was > 90%.

### Antibodies and Flow Cytometry

PBMCs isolated from fresh blood or cultured cells were stained with the following mouse anti-human antibodies: PerCP-Cy5.5-conjugated CD3, BV605 CD3, BV605 CD56, FITC CD56, PE TRAIL, PE NKP30, APC KIR3DL1, APC NKP46, APC CD244, FITC CD4, APC CD25, APC GranzymeB, FITC IFN-γ, PE TNF, and PE IL-10 (BD Biosciences); PE NKG2A, APC NKG2A, and FITC NKG2A (Miltenyi Biotec), and Alexa Flour 660 FoxP3 (eBioscience), and FITC Eomes (Thermo Fisher Scientific). A BD FACSCanto flow cytometer was used to assess stained cells and data were analyzed using Flowjo software VX (TreeStar, United States).

To analyze intracellular IFN-γ and IL-10 secretion, cells were stimulated with medium containing 10% fetal bovine serum (FBS; Gibco) with 50 ng/ml phorbol 12-myristate 13-acetate (Sigma-Aldrich), 50 ng/ml ionomycin (Merck Millipore), and 50 ng/ml monensin (Sigma-Aldrich), concurrently for 4 h. Then, cells were fixed and then permeabilized and stained with FITC mouse anti-human IFN-γ or PE mouse anti-human IL-10.

### Cytometric Bead Array

Cytokines in serum and culture supernatant samples were analyzed by cytometric bead array (CBA) using a human Th1/Th2 Cytokine Kit II (BD Biosciences, 551809). Samples (50 μl) or standard recombinant protein dilutions were added to a mixture of cytokine beads (IL- 2, IL-4, IL-6, IL-10, TNF, and IFN-γ) and PE-conjugated detection reagent. After 3 h, capture beads were washed using CBA buffer and detected by flow cytometry (BD FACSCalibur), and cytokine concentrations quantified for each sample using recombinant standards and analysis software.

### Serological Testing

Serum ALT was determined using an automatic biochemical analyzer (Cobas 8000, Roche Diagnostics GmbH, Switzerland). For the measurement of HBsAg and HBeAg, samples were analyzed using commercial enzyme immunoassay kits (Zhongshan Bio-Tech, China). The serum HBV DNA level was quantified using a real-time PCR machine (Roche LightCycler480, Switzerland). The above indicators were measured in The First Affiliated Hospital of Anhui Medical University.

### *In vitro* Culture Systems

#### PBMC Culture System

A total of 2 × 10^5^ PBMCs from patients with CHB were cultured in DMEM (HyClone SH30022.01) supplemented with 10% FBS and IL-2 (100 IU/ml), in the presence of an anti-human NKG2A blocking antibody (CloneZ199, Beckman Coulter, United States) or control IgG (BD Biosciences) at 37°C in 24-well plates. After 7 days, the phenotype and function of NK cells were analyzed by flow cytometry.

PBMCs (2 × 10^5^) isolated from healthy donors were seeded into 24-well plates in DMEM in 20% serum from healthy controls containing 100 IU/ml IL-2, then 500 ng/ml HBeAg (Prospec, HBV272) was added into the wells and cells were cultured for 7 days at 37°C. In the presence of HBeAg, 50 ng/ml anti-human IL-10 neutralizing antibody (Clone25209, R&D, United States) or control IgG (BD Biosciences) was added. The intracellular cytokine in NK cells and the cytokine secreted in the supernatant were measured by flow cytometry.

#### Co-culture System

NK cells (5 × 10^4^) and autologous CD4^+^CD25^+^ Tregs (5 × 10^4^) purified from healthy donors were cultured with 20% serum from patients with CHB and 50 ng/ml anti-IL-10 neutralizing antibody or control IgG in DMEM containing 100 IU/ml IL-2 for 3 days at 37°C in 96-well plates_._ The cytokine in the supernatant was measured by flow cytometry after culture.

Purified NK cells (5 × 10^4^) and autologous Tregs (5 × 10^4^) from healthy controls at a 1:1 ratio were co-cultured in DMEM supplemented with 10% FBS and IL-2 (100 IU/ml), with or without 500 ng/ml HBeAg and 50 ng/ml anti-IL-10 or control IgG, in the presence of 50 ng/ml anti-CD3 and 50 ng/ml anti-CD28 (BD Bioscience) for 3 days. NK cells were stained for intracellular expression of IFN-γ and the expression of cytokines in culture supernatant measured by CBA analysis. Cells were cultured in 96-well plates.

### Statistical Analysis

All data are expressed as the mean ± SEM and were analyzed using GraphPad Prism 5.0 software. The independent samples *t*-test was used to evaluate quantitative variables. Correlation analysis was by Pearson analysis. Significant differences were defined as *P* < 0.05.

## Results

### Increased Expression of the Inhibitory Receptor, NKG2A, on Circulating NK Cells in Patients With CHB Infection

**FIGURE 1:**
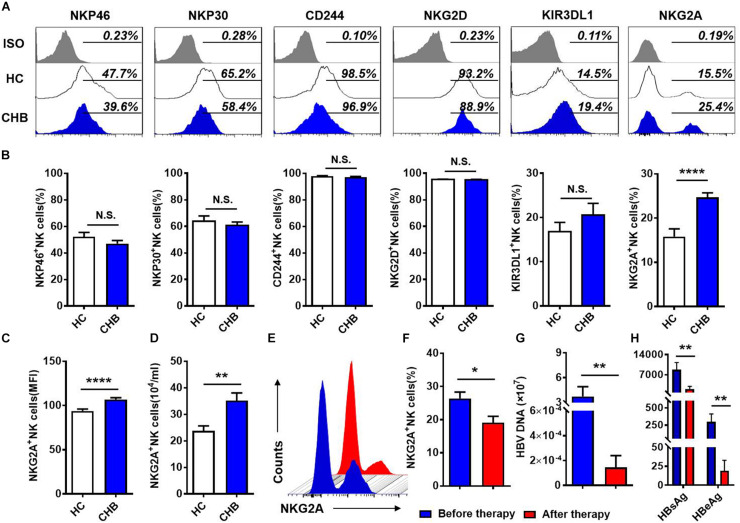
NKG2A expression is increased on circulating NK cells in patients with CHB. **(A)** NKG2A expression is increased on circulating NK cells in patients with CHB. **(A)** Representative flow cytometry plots showing expression of NKG2A, KIR3DL1, NKP46, NKP30, CD244, NKG2D, and their isotype controls on peripheral NK cells from patients with CHB and healthy controls. **(B)** Comparison of the percentages of receptors in **(A)** expressed on peripheral NK cells in patients with CHB. **(C)** Mean fluorescence intensity (MFI) of NKG2A expression on peripheral NK cells in patients with CHB and healthy controls. **(D)** Absolute numbers of NKG2A + NK cells in peripheral blood samples from patients with CHB and healthy controls. z Representative flow cytometry plots showing NKG2A expression on peripheral NK cells from patients with CHB before and after antiviral therapy. **(F)** The percentage of NKG2A + NK cells in CHB patients before therapy and after therapy. **(G)** The HBV-DNA titers of CHB patients before therapy and after therapy. **(H)** The serum HBsAg and HBeAg levels of CHB patients before therapy and after therapy. Data are representative of more than three independent experiments. Results are presented as the mean ± SEM (*n* ≥ 3 per group) and unpaired/paired two-tailed Student’s *t*-tests were conducted; *p < 0.05, **p < 0.01, ****p < 0.0001; N.S., not significant.

To investigate the NK cell phenotype, we analyzed the expression of activating (NKP30, NKP46, NKG2D, and CD244) and inhibitory (KIR3DL1, NKG2A) receptors on NK cells in the peripheral blood of patients with CHB and healthy controls, respectively. Gating strategies of lymphocytes and NK cells were showed in Supplementary Figure S1. Expression of inhibitory receptor, NKG2A, was significantly increased in patients with CHB compared with healthy donors, while there was no difference in the expression of other NK cell receptors, including KIR3DL1, NKP46, NKP30, CD244, and NKG2D (Figures 1A,B) between patients with CHB and healthy controls. Furthermore, both the mean fluorescence intensity and the absolute number of NKG2A^+^ NK cells were higher in peripheral blood from patients with CHB relative to healthy controls (Figures 1C,D), however, there was no significant difference in NKG2A expression on CD8^+^ T cells in patients with CHB compared with healthy controls (Supplementary Figures S2A,B). Moreover, in patients with CHB who had received effective antiviral treatment (entecavir, 0.5 mg per day for 6 months), NKG2A levels on NK cells were significantly reduced (Figures 1E,F), and serum levels of HBV-DNA, HBsAg, and HBeAg were also significantly reduced after therapy (Figures 1G,H).

**FIGURE 2:**
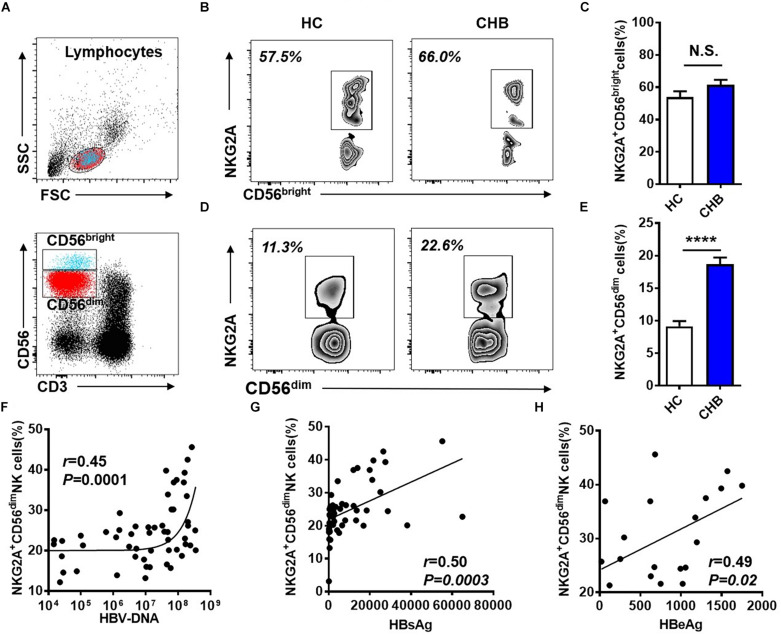
Upregulation of NKG2A on CD56^dim^ NK cells and its positive correlation with HBV DNA levels. **(A)** Sequential strategy for gating CD56^dim^ and CD56^bright^ NK cells from lymphocytes with monitoring via flow cytometry. **(B,C)** Representative data plots show NKG2A expression on CD56^dim^ and CD56^bright^ NK cells in healthy controls and patients with CHB. **(D)** NKG2A^+^CD56^bright^ NK cells and **(E)** NKG2A^+^CD56^dim^ NK cells were detected in peripheral blood samples from patients with CHB and healthy controls. **(F)** Correlation between the percentage of NKG2A^+^CD56^dim^ NK cells and serum HBV-DNA levels in patients with CHB. **(G)** Correlation between the percentage of NKG2A^+^CD56^dim^ NK cells and serum HBsAg levels in patients with CHB. **(H)** Correlation between the percentage of NKG2A^+^CD56^dim^ NK cells and serum HBeAg levels in patients with CHB. Data are representative of more than three independent experiments. Results are presented as the mean ± SEM (*n* ≥ 3 per group) and unpaired two-tailed Student’s *t-*tests or Spearman’s correlation coefficients were used for analyses; ****p < 0.0001; N.S., not significant.

NK cells can be divided into two subpopulations, CD56^dim^ and CD56^bright^, and we further investigated NKG2A expression on these two subsets in peripheral blood from patients with CHB. The gating strategies to separate CD56^dim^ and CD56^bright^ NK cells from PBMCs are detailed in Figure 2A. The frequency of NKG2A^+^ cells among the CD56^bright^ NK subpopulation did not differ significantly between these two groups (Figures 2B,C), while the. percentage of NKG2A^+^ cells among the CD56^dim^ NK subpopulation in patients with CHB was significantly higher than that in healthy controls (Figures 2D,E). Furthermore, serum HBV-DNA was found to positively correlate with the percentage of NKG2A^+^CD56^dim^ NK cells in CHB patients (*r* = 0.45, *p* = 0.0001, Figure 2F). In addition, serum levels of HBsAg and HBeAg were found to be positively associated with the percentage of NKG2A^+^CD56^dim^ NK cells (*r* = 0.50, *p* = 0.0003; *r* = 0.49, *p* = 0.03, Figures 2G,H), while there was no significant correlation between NKG2A^+^CD56^dim^ NK cells and the level of transaminases in patients with CHB (Supplementary Figures S3A,B).

TNF-related apoptosis-inducing ligand (TRAIL) is expressed by NK cell and it contributes to the elimination of HBV-specific CD8^+^ T cells in CHB (Peppa et al., 2013). We found that expression of TRAIL was upregulated on NK cells in patients with CHB, however, the frequency of TRAIL^+^ NK cells did not significantly correlate with HBV-DNA in patients with CHB infection (Supplementary Figures S4A–C). These results indicate that chronic HBV infection induces an increased expression of NKG2A on CD56^dim^ NK cells, and that upregulation of NKG2A may be linked with the disease progression in CHB.

### Blocking NKG2A Restores the Ability of NK Cells From Patients With CHB to Produce Cytokines

**FIGURE 3:**
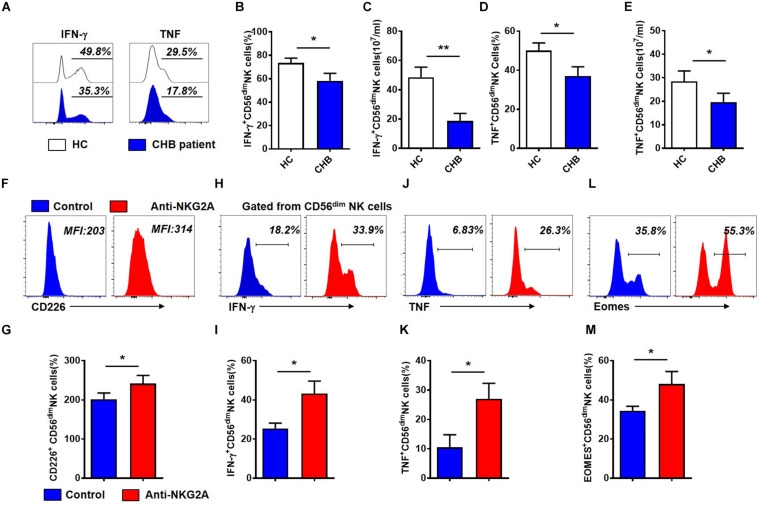
Cytokine production by CD56^dim^ NK cells is restored after blockade of NKG2A signaling in patients with CHB**. (A)** Representative flow cytometry plots showing IFN- γ and TNF expression in NK cells from patients with CHB and healthy controls. **(B)** Analysis of IFN- γ ^+^CD56^dim^ NK cells in patients with CHB and healthy controls. **(C)** Comparison of absolute numbers of IFN - γ ^+^ CD56^dim^ NK cells in patients with CHB and healthy controls. **(D)** Analysis of TNF^+^CD56^dim^ NK cells in patients with CHB and healthy controls. **(E)** The absolute number of TNF^+^CD56^dim^ NK cells in patients with CHB and healthy controls. **(F–M)** NK cells isolated from patients with CHB were cultured in DMEM supplemented with 10% FBS and 100 IU/ml IL-2, with anti-human NKG2A antibody or control IgG. After 3 days, the phenotype and function of NK cells were analyzed by flow cytometry. **(F)** Representative plots of NKG2A and CD226 expressed in CD56^dim^ NK cells after NK cells were cultured with anti-human NKG2A antibody or control IgG. **(G)** Expression of CD226 on total NK cells, CD56^bright^ and CD56^dim^ NK cells after isolated CHB NK cells were cultured with anti-human NKG2A antibody or control IgG. **(H)** Representative plots of IFN- γ expressed in CD56^dim^ NK cells after NK cells were cultured with anti-human NKG2A antibody or control IgG. **(I)** Expression of IFN- γ on total NK cells, CD56^bright^ and CD56^dim^ NK cells after isolated CHB NK cells were cultured with anti-human NKG2A antibody or control IgG. **(J)** Representative plots of TNF expressed in CD56^dim^ NK cells after NK cells were cultured with anti-human NKG2A antibody or control IgG. **(K)** Expression of TNF on total NK cells, CD56bright and CD56^dim^ NK cells after isolated CHB NK cells were cultured with anti-human NKG2A antibody or control IgG. **(L)** Representative plots of Eomes expressed in CD56^dim^ NK cells after NK cells were cultured with anti-human NKG2A antibody or control IgG. **(M)** Expression of Eomes on total NK cells, CD56^brigh^t and CD56^dim^ NK cells after isolated CHB NK cells were cultured with anti-human NKG2A antibody or control IgG. Results are presented as the mean ± SEM (*n* ≥ 3 per group) and unpaired/paired two-tailed Student’s *t*-tests were conducted; *p < 0.05, **p < 0.01. N.S., not significant.

To evaluate the impact of increased NKG2A expression on NK cells in patients with CHB, we next assessed the function of NK cells from these patients. Gating strategies of lymphocytes, NK cells and CD56^dim^ NK cells were showed in Supplementary Figure S5. As shown in Figure 3A, the percentage and absolute numbers of IFN-γ^+^CD56^dim^ NK cells were lower in patients with CHB than those in healthy controls (Figures 3B,C). Moreover, the frequency and absolute number of TNF^+^CD56^dim^ NK cells were also significantly decreased in patients with CHB relative to controls (Figures 3D,E). These data demonstrate that cytokine production is impaired in NK cells from patients with CHB relative to healthy controls.

Next, to investigate the role of NKG2A in NK cell function, we purified NK cells from patients with CHB and incubated them with anti-human NKG2A blocking antibody *in vitro* (Supplementary Figure S6B). Gating information of CD56^dim^ NK cells was showed in Supplementary Figure S6A. Our results showed that blocking NKG2A significantly enhanced expression of the activating receptor, CD226, on CD56^dim^ NK cells isolated from patients with active CHB (Figures 3F,G), and significantly increased the expression of IFN-γ (Figures 3H,I) as well as TNF in CD56^dim^ NK cells (Figures 3J,K). Meanwhile, the frequency of Eomes^+^CD56^dim^ NK cells was also significantly enhanced when NKG2A was blocked (Figures 3L,M). While the expression of CD226, IFN-γ, TNF and Eomes on NK cells were significantly increased in NKG2A blockade group compared with control, but there was no significant difference in the expression of these four markers on CD56^bright^ NK cells between these two groups (Supplementary Figures S6C–F). In addition, the expression of TRAIL and Granzyme-B did not change significantly in this assay (*P* > 0.05, Supplementary Figures S6G,H). These results suggest that the inhibitory receptor, NKG2A, expressed on NK cells, accounts for the impaired ability of NK cells to produce cytokines in patients with CHB infection, and that blocking NKG2A *in vitro* can restore the function of those NK cells.

### IL-10^+^ Tregs Contribute to the Dysfunction of NKG2A^+^ NK Cells in Patients With CHB

**FIGURE 4:**
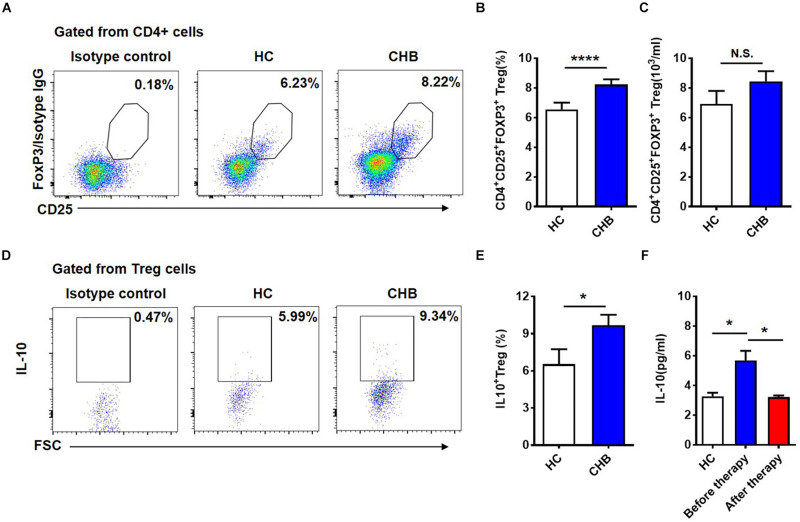
Circulating Tregs and secreted IL-10 in patients with CHB infection. **(A)** Representative flow cytometry plots showing CD4^+^CD25^+^FOXP3^+^ Tregs in peripheral blood samples from patients with CHB and healthy controls. **(B)** Data from **(A)** were compiled and analyzed for significance using the *t*-test. **(C)** Comparison of the absolutely numbers of CD4^+^CD25^+^FOXP3^+^ Tregs in peripheral blood samples from patients with CHB and healthy controls. **(D)** Representative flow cytometry plots of IL-10^+^ Tregs in peripheral blood samples from patients with CHB and healthy controls. **(E)** Data from **(D)** were compiled and analyzed for significance using the *t*-test. **(F)** Comparison of serum IL-10 levels detected using a CBA kit in healthy controls, and patients with CHB patients before and after antiviral treatment. Data are representative of more than three independent experiments. Results are presented as the mean ± SEM (*n* ≥ 3 per group) and unpaired two-tailed Student’s *t*-tests and one-way ANOVA were conducted; *p < 0.05, ****p < 0.0001. N.S., not significant.

We wondered which factors were responsible for the induction of NKG2A expression on NK cells. First, we examined CD4^+^CD25^+^Foxp3^+^ Tregs in patients with CHB and found that both the proportion and absolute number of Tregs in peripheral blood were significantly higher in patients with CHB than those in healthy controls (Figures 4A–C). Next, we measured cytokine production in Tregs and found that Tregs from CHB patients produced much higher levels than those from healthy controls (Figures 4D,E). Further, we assessed the level of serum IL-10 in healthy controls and CHB patients who had received effective antiviral therapy, and found that serum IL-10 level in treated CHB patients returned to a normal level, which was significantly lower than that in patients with CHB who had not received effective therapy (Figure 4F).

**FIGURE 5:**
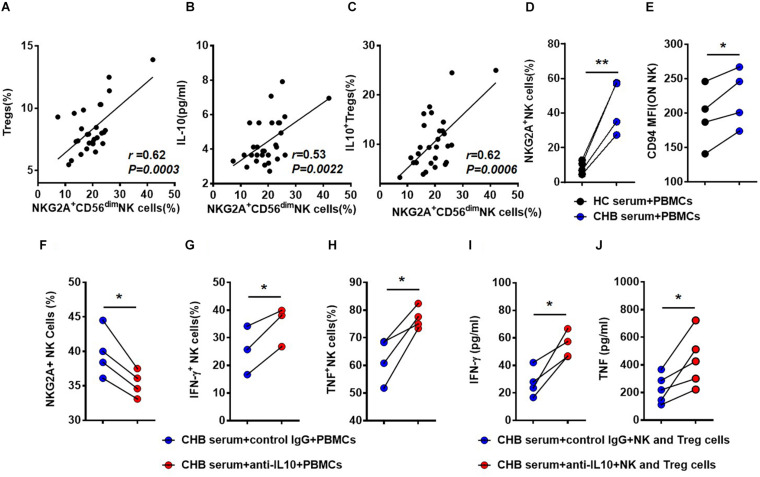
Treg-derived IL-10 impairs NK cell function in patients with CHB. **(A)** Correlation between the percentage of NKG2A^+^CD56^dim^ NK cells and Tregs in patients with CHB determined by flow cytometry. **(B)** Correlation between the percentage of NKG2A^+^CD56^dim^ NK cells and serum IL-10 levels in serum samples from patients with CHB patients. **(C)** Correlation between the percentage of NKG2A^+^CD56^dim^ NK cells and IL-10^+^ Tregs in peripheral blood samples from patients with CHB. **(D,E)** PBMCs from healthy donors were cultured with 100 IU/ml IL-2 in the presence of 20% CHB or healthy control serum for 7 days. After 7 days of culture in the presence of 20% CHB or healthy control serum, expression of NKG2A and CD94 was monitored on NK cells by flow cytometry. **(F–H)** PBMCs from healthy donors were cultured with 20% CHB and 100 IU/ml IL-2 in the presence of 50 ng/ml anti-human IL-10 neutralizing antibody or control IgG. After 7 days, the percentages of NKG2A^+^ NK cells, IFN- γ^+^ NK cells and TNF^+^ NK cells were detected after treatment of PBMCs with CHB serum and anti-IL-10 or control IgG *in vitro*. **(I,J)** NK cells (5 × 104) and Tregs (5 × 104) were purified from healthy donors and co-cultured in the presence of 20% CHB patient serum and 100 IU/ml IL-2, supplemented with anti-CD3 and-CD28 mAb, with 50 ng/ml anti-human IL-10 neutralizing antibody for 3 days. **(I)** The concentration of IFN-γ in the supernatant was measured using a CBA kit. **(J)** The concentration of TNF in the supernatant was measured using a CBA kit. Data are representative of more than three independent experiments. Results are presented as the mean ± SEM (*n* ≥ 3 per group) and paired two-tailed Student’s *t*-tests or Spearman’s correlation coefficients were conducted; *p < 0.05; **p < 0.01.

Interestingly, we detected a positive correlation between the percentage of Tregs and that of NKG2A^+^CD56^dim^ NK cells (Figure 5A). And both the percentage of IL-10^+^ Tregs and serum IL-10 level positively correlated with the percentage of NKG2A^+^CD56^dim^ NK cells (Figures 5B,C). To evaluate the effects of IL-10 on NKG2A expression, PBMCs were isolated from healthy donors and stimulated with 20% sera from CHB patients. We observed that the percentages of NKG2A^+^ and CD94^+^ NK cells were significantly increased by stimulation with CHB sera (Figures 5D,E). Interestingly, the percentage of NKG2A^+^ NK cells was significantly reduced in PBMCs stimulated with CHB sera in the presence of anti-IL-10, relative to those stimulated with CHB sera only (Figure 5F). Furthermore, the percentage of NKG2A^+^ NK cells was lower accompanied by a higher frequency of IFN-γ ^+^ and TNF^+^ NK cells in the IL-10 blockade group when compared to that in the control group (Figures 5G,H). Accordingly, when NK cells and Tregs purified from healthy controls were stimulated with anti-CD3 and anti-CD28, and cultured with anti-human IL-10 neutralizing antibody or control IgG in the presence of CHB sera, we found that the concentrations of IFN-γ and TNF in the culture supernatant were significantly increased after IL-10 blockade (Figures 5I,J). These findings suggest that Treg-derived IL-10 is involved in induction of NKG2A^+^ NK cell and NK cell dysfunction.

### HBeAg Induces NKG2A+ NK Cell Dysfunction Mediated by Treg-Derived IL-10

**FIGURE 6:**
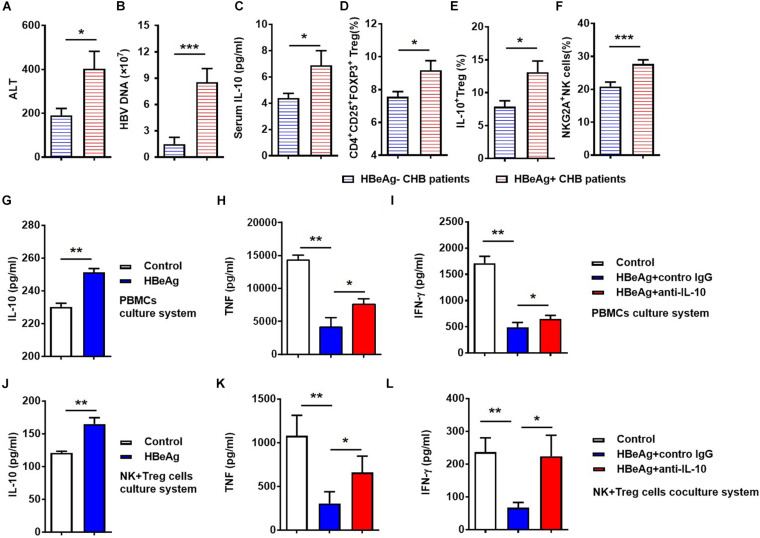
HBeAg-induced IL-10 suppresses NK cell activation *in vitro*. **(A)** Serum ALT levels, **(B)** serum HBV DNA copy numbers, **(C)** serum IL-10 levels, **(D)** CD56^+^NKG2A^+^ NK expression, **(E)** percentages of Tregs, and **(F)** percentages of IL-10^+^ Tregs were compared in HBeAg-positive and -negative patients with CHB. **(G–I)** For vitro experiments, PBMCs (2 × 10^5^) isolated from healthy donors were cultured with or without 500 ng/ml HBeAg and 50 ng/ml anti-human IL-10 neutralizing antibody or control IgG in the presence of 100 IU/ml IL-2 for 7 days and the concentrations of cytokines in culture supernatant measured using a CBA kit. **(G)** IL-10 levels in supernatant after culture of PBMCs from healthy donors with or without HBeAg. **(H,I)** IFN-γ and TNF levels in supernatant after culture of PBMCs with HBeAg, in the presence of anti-IL-10 or control IgG. **(J–L)** In the co-culture system, 5 × 10^4^ NK cells and 5 × 10^4^ Tregs were purified from healthy donors and then co-cultured in the presence of 10% FBS, anti-CD3, and -CD28 mAb, with or without 500 ng/ml HBeAg and 50 ng/ml anti-IL-10 or control IgG in the presence of 100 IU/ml IL-2 for 3 days, then the concentration of cytokines in supernatant were measured using a CBA kit. **(J)** IL-10 in culture supernatants after co-culture of NK cells and Tregs with or without HBeAg for 3 days. **(K,L)** IFN-γ and TNF levels were detected in culture supernatant after co-culture of NK cells and Tregs with HBeAg in the presence of anti-IL-10 or control IgG. Data are representative of more than three independent experiments. Results are presented as the mean ± SEM (*n* ≥ 3 per group) and unpaired/paired two-tailed Student’s *t-*tests were conducted; *p < 0.05, **p < 0.01, ***p < 0.001.

HBeAg has been suggested to play an important role in maintaining HBV persistence in patients with CHB. We next compared serum ALT and HBV-DNA between HBeAg-positive and -negative patients, and found that ALT and HBV-DNA levels were higher in HBeAg-positive patients than those that are HBeAg-negative (Figures 6A,B). Specifically, we observed that HBeAg-positive patients had significantly increased serum level of IL-10, as well as increased percentages of total Tregs, IL-10^+^ Tregs and NKG2A^+^ NK cells, respectively (Figures 6C–F). Together, these results suggest that HBeAg is associated with Treg-derived IL-10 production and NKG2A expression on NK cells in CHB patients.

To delineate the effect of HBeAg on NKG2A^+^ NK cell dysfunction in patients with CHB, PBMCs were isolated from healthy controls and stimulated with HBeAg *in vitro*. We found that HBeAg induced human PBMCs to produce significantly more IL-10 upon stimulation with PMA, ionomycin, and monensin *in vitro* (Figure 6G). Moreover, levels of TNF and IFN-γ were decreased in PBMCs stimulated with HBeAg, while anti-IL-10 treatment increased the levels of these cytokines in the culture supernatant (Figures 6H,I). To test the direct effect of HBeAg on regulating NK cells, we added HBeAg directly to NK cells alone, but found that the frequency of NKG2A^+^ NK cells did not alter significantly (Supplementary Figure S7). To directly investigate the possible role of HBeAg in regulating Treg and NK cells, these cells were purified from healthy controls and co-cultured *in vitro*. Addition of HBeAg to the culture system in the presence of anti-CD3 and anti-CD28 led to increased amounts of IL-10 in the supernatant (Figure 6J), accompanied by reduced levels of TNF and IFN-γ (Figures 6K,L); the defective production of these two cytokines was restored by addition of anti-IL-10 (Figures 6K,L). Together, these findings indicate that HBeAg promotes IL-10 production by Tregs, thereby inducing NKG2A expression on NK cells and contributing to the impaired cytokine-produced ability of NK cells.

## Discussion

**FIGURE 7:**
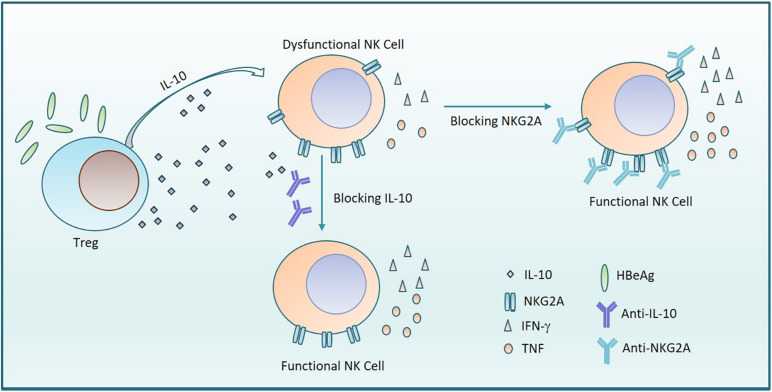
HBeAg induces NKG2A^+^ NK cell dysfunction via Tregs-derived IL-10. The diagram shows that HBeAg induces NK cell dysfunction with upregulated expression of NKG2A on NK cells and reduced secretion of TNF and IFN- γ, which is mediated by increased amounts of IL-10 secreted from Tregs. On the one hand, blocking NKG2A on NK cells leads to the recovery of NK cell function. On the other hand, the function of NK cells is also restored by addition of anti-IL-10.

CHB infection-induced immune tolerance is the biggest obstacle to the elimination of HBV in the host. As the vital effector lymphocytes of innate immune system, NK cells have an important role in defending against viruses and tumors, via rapid cytotoxic activity and cytokine production. In this study, we demonstrate that the percentage of NKG2A^+^ NK cells increased in patients with CHB. We found that NKG2A^+^CD56^dim^ NK cells correlated with HBV infection and NKG2A blockade could restore the function of NK cells *in vitro*. Furthermore, IL-10^+^ Treg cells increased in patients with CHB and the frequency of NKG2A^+^ NK cells was positively associated with Treg-derived IL-10, which is likely to contribute to the induction of NKG2A^+^ NK cell dysfunction in CHB patients. Moreover, we found that the frequency of NKG2A^+^ NK cell and level of Treg-derived IL-10 were elevated in HBeAg-positive patients relative to HBeAg-negative patients and that HBeAg promoted IL-10 production by Tregs, which further contributed to increased NKG2A expression on NK cells resulting in NK cell dysfunction (Figure 7).

NK cells are critical for HBV clearance in an HBV-transfected mouse model mimicking acute HBV infection in patients (Zheng et al., 2016). The expression pattern of various receptors on NK cells is abnormal, leading to their dysfunction during CHB infection (Sun et al., 2012; Peng and Tian, 2018). Down-regulation of activating receptors and up-regulation of inhibitory receptors on NK cells are associated with increased HBV viral load (Tjwa et al., 2011). NKG2A is also important for the maintenance of persistent hepatitis C virus infection (Golden-Mason et al., 2008). In patients with HCC, NKG2A, a checkpoint candidate, is expressed on NK cells and mediates NK cell dysfunction in intratumor tissues (Sun et al., 2017). Li et al. (2013) found that NKG2A expression is higher in patients with CHB and that, in a mouse model established by transfection of HBV plasmid, blocking NKG2A signaling promotes viral clearance. These data are consistent with our finding that up-regulation of NKG2A on CD56^dim^ NK is positively associated with the serum levels of HBV-DNA, HBsAg, and HBeAg in patients with CHB (Figure 2). We also demonstrated that NKG2A blockade restores the function of CD56^dim^ NK cells; specifically, this blockade enhances the IFN-γ and TNF production, and induces the expression of CD226 and Eomes in CD56^dim^ NK cells *in vitro* (Figure 3). Furthermore, NKG2A is expressed on CD8^+^ T cells (Moser et al., 2002), however, no significant expression of NKG2A was detected on CD8^+^ T cells in CHB patients in our study (Supplementary Figure S1). As NK cell dysfunction is associated with impaired CD8^+^ T cell responses in liver disease (Li et al., 2018; Zheng and Tian, 2019), it is possible that the elevated expression of NKG2A on NK cells is linked with dysfunctional CD8^+^ T cells in patients with CHB.

In addition, after 24-week tenofovir treatment, the percentage of NKG2A^+^ NK cells transiently decreased (Lv et al., 2012). Similarly, following treatment with PEG-interferon alpha-2a and adefovir for 48 weeks, the cytotoxic function of NK cells was restored and their IFN-γ secretion increased, while the number of NKG2A^+^ NK cells was notably down-regulated (de Niet et al., 2017). In this study, we found that the frequency of NKG2A^+^ NK cells, serum level of IL-10, and the levels of HBV-DNA, HBeAg, and HBsAg were significantly reduced after antiviral therapy (Figures 1E–G). IL-10 has an important role in sustaining the expression of NKG2A^+^Ly49^–^ on hepatic NK cells (Lassen et al., 2010). In an HBV-transfected mouse model, Li et al. demonstrated that Treg-derived IL-10 contributes to the upregulation of NKG2A expression on NK cells (Li et al., 2013). In liver diseases (e.g., CHB, cirrhosis and liver cancer), NK cell dysfunction is induced by elevated levels of IL-10 and TGF-β (Lunemann et al., 2014; Yang et al., 2017). The production of IL-10 is significantly enhanced by increased HBV titers, and IL-10 may suppress IFN- γ production in NK cells in patients with CHB (Li et al., 2010; Peppa et al., 2010; Tan et al., 2010). Our data show that Tregs increased the percentage of NKG2A cells in the presence of CHB serum supplemented with anti-CD3 and CD28, while secretion of IFN-γ and TNF from NK cells was up-regulated by IL-10 blockade in this co-culture system (Figure 5).

HBeAg positivity is associated with viral replication and immunotolerance in CHB infection (European Association for the Study of The Liver, 2012). Moreover, CD4^+^CD25^+^ T cells are significantly elevated in HBeAg-positive patients when compared to HBeAg-negative patients, and their number is positively correlated with HBeAg levels (El-Badawy et al., 2012). HBeAg evades host immune responses by inhibiting lipopolysaccharide-induced NLRP3 inflammasome activation, which is critical for antiviral defense (Yu et al., 2017b). Loss of HBeAg is associated with short-term evolution, as it results in loss of HBeAg-mediated tolerance and reduced transmissibility of the HBeAg(-) virion (Kramvis et al., 2018). Furthermore, maternal-derived HBeAg was reported to contribute to the impaired function of hepatic macrophage cells (Tian et al., 2016). Consistently, HBeAg-induced expansion of monocytic myeloid-derived suppressor cells leads to impaired CD8^+^ T cell responses in CHB infection (Yang et al., 2019). Moreover, the frequency and skewed T-cell receptor beta-chain variable patterns of peripheral Tregs correlate with HBeAg seroconversion (Yang et al., 2016), and CD3^+^CD4^+^Foxp3^–^ cells can be induced to convert into CD3^+^CD4^+^Foxp3^+^ Tregs in liver-draining lymph nodes (Yu et al., 2017a). There are also reports indicating that liver sinusoidal endothelial cells and B cells contribute to Treg induction (Carambia et al., 2014; Lu et al., 2015).

In our study, we demonstrate that HBeAg-positive patients had higher frequencies of NKG2A^+^ NK cells than those who were HBeAg-negative. Tregs and IL-10 derived from these cells were significantly elevated in HBeAg-positive patients. When PBMCs or NK cells and Tregs purified from healthy controls were co-cultured with anti-CD3 and anti-CD28 *in vitro*, the addition of HBeAg led to increased production of IL-10 accompanied by reduced levels of TNF and IFN-γ, while the addition of HBeAg to NK cells alone did not have significant impact on the function of NK cells. Furthermore, the production of TNF and IFN-γ was restored by IL-10 blockade. These results indicate that HBeAg contributes to the up-regulation of IL-10^+^ Tregs, then causing NK cell dysfunction (Figure 6).

In summary, during CHB infection, HBeAg is associated with HBV immune tolerance. We found that HBeAg induces IL-10 production in Tregs, which subsequently upregulates the expression of NKG2A on NK cells, leading to NK cell dysfunction, suggesting that HBeAg accounts for NK cell dysfunction in CHB patients. Together, our findings contribute to the understanding of the mechanisms underlying NK cell dysfunction in CHB patients and indicate that the HBeAg-IL-10-NKG2A^+^ NK cell axis is a potential therapeutic target in these patients.

## Data Availability Statement

The datasets generated for this study are available on request to the corresponding author.

## Ethics Statement

The studies involving human participants were reviewed and approved by the local ethics committee of The First Affiliated Hospital of Anhui Medical University and the local ethics committee of Chaohu Hospital of Anhui Medical University. The patients/participants provided their written informed consent to participate in this study.

## Author Contributions

QM and XD designed and wrote the manuscript. QM, XD, SL, and DS performed the experiments and analyses. TZ, CZ, QL, MiZ, YY, and JC were involved in the collection of clinical samples. LZ and YX critically reviewed the manuscript. YG supplied and evaluated CHB patients. MeZ designed and supervised the experiments and wrote the manuscript.

## Conflict of Interest

The authors declare that the research was conducted in the absence of any commercial or financial relationships that could be construed as a potential conflict of interest.

## References

[B1] Carambia A. Freund B. Schwinge D. Heine M. Laschtowitz A. Huber S. (2014). TGF-beta-dependent induction of CD4(+)CD25(+)Foxp3(+) Tregs by liver sinusoidal endothelial cells. *J. Hepatol.* 61 594–599. 10.1016/j.jhep.2014.04.027 24798620

[B2] de Niet A. Jansen L. Stelma F. Willemse S. B. Kuiken S. D. Weijer S. (2017). Peg-interferon plus nucleotide analogue treatment versus no treatment in patients with chronic hepatitis B with a low viral load: a randomised controlled, open-label trial. *Lancet Gastroenterol. Hepatol.* 2 576–584. 10.1016/S2468-1253(17)30083-3 28522204

[B3] Dienstag J. L. (2008). Hepatitis B virus infection. *N. Engl. J. Med.* 359 1486–1500. 18832247 10.1056/NEJMra0801644

[B4] El-Badawy O. Sayed D. Badary M. S. Abd-Alrahman M. E. El-Feky M. A. Thabit A. G. (2012). Relations of regulatory T cells with hepatitis markers in chronic hepatitis B virus infection. *Hum. Immunol.* 73 335–341. 10.1016/j.humimm.2012.01.014 22342871

[B5] El-Deeb N. M. El-Adawi H. I. El-Wahab A. E. A. Haddad A. M. El Enshasy H. A. He Y. W. (2019). Modulation of NKG2D, KIR2DL and cytokine production by pleurotus ostreatus glucan enhances natural killer cell cytotoxicity toward cancer cells. *Front. Cell Dev. Biol.* 7:165. 10.3389/fcell.2019.00165 31457012 PMC6700253

[B6] European Association for the Study of The Liver, (2012). EASL clinical practice guidelines: management of chronic hepatitis B virus infection. *J. Hepatol.* 57 167–185. 22436845 10.1016/j.jhep.2012.02.010

[B7] Golden-Mason L. Bambha K. M. Cheng L. Howell C. D. Taylor M. W. Clark P. J. (2011). Natural killer inhibitory receptor expression associated with treatment failure and interleukin-28B genotype in patients with chronic hepatitis C. *Hepatology* 54 1559–1569. 10.1002/hep.24556 21983945 PMC3206734

[B8] Golden-Mason L. Madrigal-Estebas L. Mcgrath E. Conroy M. J. Ryan E. J. Hegarty J. E. (2008). Altered natural killer cell subset distributions in resolved and persistent hepatitis C virus infection following single source exposure. *Gut* 57 1121–1128. 10.1136/gut.2007.130963 18372499

[B9] Haanen J. B. Cerundolo V. (2018). NKG2A, a new kid on the immune checkpoint block. *Cell* 175 1720–1722. 10.1016/j.cell.2018.11.048 30550781

[B10] Kramvis A. Kostaki E. G. Hatzakis A. Paraskevis D. (2018). Immunomodulatory function of HBeAg related to short-sighted evolution, transmissibility, and clinical manifestation of hepatitis B virus. *Front. Microbiol.* 9:2521. 10.3389/fmicb.2018.02521 30405578 PMC6207641

[B11] Lassen M. G. Lukens J. R. Dolina J. S. Brown M. G. Hahn Y. S. (2010). Intrahepatic IL-10 maintains NKG2A+Ly49- liver NK cells in a functionally hyporesponsive state. *J. Immunol.* 184 2693–2701. 10.4049/jimmunol.0901362 20124099 PMC2885840

[B12] Li F. Wei H. Wei H. Gao Y. Xu L. Yin W. (2013). Blocking the natural killer cell inhibitory receptor NKG2A increases activity of human natural killer cells and clears hepatitis B virus infection in mice. *Gastroenterology* 144 392–401. 10.1053/j.gastro.2012.10.039 23103614

[B13] Li H. Zhai N. Wang Z. Song H. Yang Y. Cui A. (2018). Regulatory NK cells mediated between immunosuppressive monocytes and dysfunctional T cells in chronic HBV infection. *Gut* 67 2035–2044. 10.1136/gutjnl-2017-314098 28899983 PMC6176520

[B14] Li H. J. Zhai N. C. Song H. X. Yang Y. Cui A. Li T. Y. (2015). The role of immune cells in chronic HBV infection. *J. Clin. Transl. Hepatol.* 3 277–283. 10.14218/JCTH.2015.00026 26807384 PMC4721896

[B15] Li J. Wu W. Peng G. Chen F. Bai M. Zheng M. (2010). HBcAg induces interleukin-10 production, inhibiting HBcAg-specific Th17 responses in chronic hepatitis B patients. *Immunol. Cell Biol.* 88 834–841. 10.1038/icb.2010.63 20498672

[B16] Long E. O. Kim H. S. Liu D. Peterson M. E. Rajagopalan S. (2013). Controlling natural killer cell responses: integration of signals for activation and inhibition. *Annu. Rev. Immunol.* 31 227–258. 10.1146/annurev-immunol-020711-075005 23516982 PMC3868343

[B17] Lozano R. Naghavi M. Foreman K. Lim S. Shibuya K. Aboyans V. (2012). Global and regional mortality from 235 causes of death for 20 age groups in 1990 and 2010: a systematic analysis for the Global Burden of Disease Study 2010. *Lancet* 380 2095–2128. 10.1016/S0140-6736(12)61728-0 23245604 PMC10790329

[B18] Lu F. T. Yang W. Wang Y. H. Ma H. D. Tang W. Yang J. B. (2015). Thymic B cells promote thymus-derived regulatory T cell development and proliferation. *J. Autoimmun.* 61 62–72. 10.1016/j.jaut.2015.05.008 26071985

[B19] Lunemann S. Malone D. F. Hengst J. Port K. Grabowski J. Deterding K. (2014). Compromised function of natural killer cells in acute and chronic viral hepatitis. *J. Infect Dis.* 209 1362–1373. 10.1093/infdis/jit561 24154737

[B20] Lv J. Jin Q. Sun H. Chi X. Hu X. Yan H. (2012). Antiviral treatment alters the frequency of activating and inhibitory receptor-expressing natural killer cells in chronic hepatitis B virus infected patients. *Mediators Inflamm.* 2012:804043. 10.1155/2012/804043 23304062 PMC3529875

[B21] Maini M. K. Peppa D. (2013). NK cells: a double-edged sword in chronic hepatitis B virus infection. *Front. Immunol.* 4:57. 10.3389/fimmu.2013.00057 23459859 PMC3585438

[B22] Martinet J. Dufeu-Duchesne T. Bruder Costa J. Larrat S. Marlu A. Leroy V. (2012). Altered functions of plasmacytoid dendritic cells and reduced cytolytic activity of natural killer cells in patients with chronic HBV infection. *Gastroenterology* 143 1586.e8–1596.e8. 10.1053/j.gastro.2012.08.046 22960656

[B23] Moser J. M. Gibbs J. Jensen P. E. Lukacher A. E. (2002). CD94-NKG2A receptors regulate antiviral CD8(+) T cell responses. *Nat. Immunol.* 3 189–195. 10.1038/ni757 11812997

[B24] Park J. J. Wong D. K. Wahed A. S. Lee W. M. Feld J. J. Terrault N. (2016). Hepatitis B virus–specific and global t-cell dysfunction in chronic hepatitis B. *Gastroenterology* 150 684.e5–695.e5. 10.1053/j.gastro.2015.11.050 26684441 PMC4766024

[B25] Peng H. Tian Z. (2018). NK cells in liver homeostasis and viral hepatitis. *Sci. China Life Sci.* 61 1477–1485. 10.1007/s11427-018-9407-2 30421296

[B26] Peng H. Wisse E. Tian Z. (2016). Liver natural killer cells: subsets and roles in liver immunity. *Cell Mol. Immunol.* 13 328–336. 10.1038/cmi.2015.96 26639736 PMC4856807

[B27] Peppa D. Gill U. S. Reynolds G. Easom N. J. Pallett L. J. Schurich A. (2013). Up-regulation of a death receptor renders antiviral T cells susceptible to NK cell-mediated deletion. *J. Exp. Med.* 210 99–114. 10.1084/jem.20121172 23254287 PMC3549717

[B28] Peppa D. Micco L. Javaid A. Kennedy P. T. Schurich A. Dunn C. (2010). Blockade of immunosuppressive cytokines restores NK cell antiviral function in chronic hepatitis B virus infection. *PLoS Pathog.* 6:e1001227. 10.1371/journal.ppat.1001227 21187913 PMC3003000

[B29] Racanelli V. Rehermann B. (2006). The liver as an immunological organ. *Hepatology* 43 S54–S62. 16447271 10.1002/hep.21060

[B30] Rehermann B. (2013). Pathogenesis of chronic viral hepatitis: differential roles of T cells and NK cells. *Nat. Med.* 19 859–868. 10.1038/nm.3251 23836236 PMC4482132

[B31] Sun C. Fu B. Gao Y. Liao X. Sun R. Tian Z. (2012). TGF-beta1 down-regulation of NKG2D/DAP10 and 2B4/SAP expression on human NK cells contributes to HBV persistence. *PLoS Pathog.* 8:e1002594. 10.1371/journal.ppat.1002594 22438812 PMC3305436

[B32] Sun C. Xu J. Huang Q. Huang M. Wen H. Zhang C. (2017). High NKG2A expression contributes to NK cell exhaustion and predicts a poor prognosis of patients with liver cancer. *Oncoimmunology* 6:e1264562. 10.1080/2162402X.2016.1264562 28197391 PMC5283631

[B33] Tan A. T. Koh S. Goh W. Zhe H. Y. Gehring A. J. Lim S. G. (2010). A longitudinal analysis of innate and adaptive immune profile during hepatic flares in chronic hepatitis B. *J. Hepatol.* 52 330–339. 10.1016/j.jhep.2009.12.015 20137825

[B34] Tian Y. Kuo C. F. Akbari O. Ou J. H. (2016). Maternal-derived hepatitis B virus E antigen alters macrophage function in offspring to drive viral persistence after vertical transmission. *Immunity* 44 1204–1214. 10.1016/j.immuni.2016.04.008 27156385 PMC4871724

[B35] Tian Z. Chen Y. Gao B. (2013). Natural killer cells in liver disease. *Hepatology* 57 1654–1662. 23111952 10.1002/hep.26115 PMC3573257

[B36] Tjwa E. T. Van Oord G. W. Hegmans J. P. Janssen H. L. Woltman A. M. (2011). Viral load reduction improves activation and function of natural killer cells in patients with chronic hepatitis B. *J. Hepatol.* 54 209–218. 10.1016/j.jhep.2010.07.009 21095036

[B37] Trehanpati N. Vyas A. K. (2017). Immune regulation by T regulatory cells in hepatitis B virus-related inflammation and cancer. *Scand. J. Immunol.* 85 175–181. 10.1111/sji.12524 28109025

[B38] Yang F. Yu X. Zhou C. Mao R. Zhu M. Zhu H. (2019). Hepatitis B e antigen induces the expansion of monocytic myeloid-derived suppressor cells to dampen T-cell function in chronic hepatitis B virus infection. *PLoS Pathog.* 15:e1007690. 10.1371/journal.ppat.1007690 30998767 PMC6472891

[B39] Yang H. L. Zhou W. J. Chang K. K. Mei J. Huang L. Q. Wang M. Y. (2017). The crosstalk between endometrial stromal cells and macrophages impairs cytotoxicity of NK cells in endometriosis by secreting IL-10 and TGF-beta. *Reproduction* 154 815–825. 10.1530/REP-17-0342 28971893

[B40] Yang J. Sheng G. Xiao D. Shi H. Wu W. Lu H. (2016). The frequency and skewed T-cell receptor beta-chain variable patterns of peripheral CD4(+)CD25(+) regulatory T-cells are associated with hepatitis B e antigen seroconversion of chronic hepatitis B patients during antiviral treatment. *Cell Mol. Immunol.* 13 678–687. 10.1038/cmi.2015.100 26899927 PMC5037272

[B41] Yu J. Chen Y. Wu Y. Ye L. Lian Z. Wei H. (2017a). The differential organogenesis and functionality of two liver-draining lymph nodes in mice. *J. Autoimmun.* 84 109–121. 10.1016/j.jaut.2017.08.005 28886898

[B42] Yu X. Lan P. Hou X. Han Q. Lu N. Li T. (2017b). HBV inhibits LPS-induced NLRP3 inflammasome activation and IL-1beta production via suppressing the NF-kappaB pathway and ROS production. *J. Hepatol.* 66 693–702. 10.1016/j.jhep.2016.12.018 28027970

[B43] Zhang C. Wang X. M. Li S. R. Twelkmeyer T. Wang W. H. Zhang S. Y. (2019). NKG2A is a NK cell exhaustion checkpoint for HCV persistence. *Nat. Commun.* 10:1507. 10.1038/s41467-019-09212-y 30944315 PMC6447531

[B44] Zheng M. Sun H. Tian Z. (2018). Natural killer cells in liver diseases. *Front. Med.* 12 269–279. 10.1007/s11684-018-0621-4 29675689

[B45] Zheng M. Sun R. Wei H. Tian Z. (2016). NK cells help induce anti-hepatitis B virus CD8+ T cell immunity in mice. *J. Immunol.* 196 4122–4131. 10.4049/jimmunol.1500846 27183639

[B46] Zheng M. Tian Z. (2019). Liver-mediated adaptive immune tolerance. *Front. Immunol.* 10:2525. 10.3389/fimmu.2019.02525 31787967 PMC6856635

