# Supplementary material for: Hepatitis B e Antigen Induces NKG2A+ Natural Killer Cell Dysfunction via Regulatory T Cell-Derived Interleukin 10 in Chronic Hepatitis B Virus Infection
Source: Front Cell Dev Biol. 2020 Jun 3;8:421. doi: 10.3389/fcell.2020.00421 (PMC7283553; doi:10.3389/fcell.2020.00421)
Supplement: Supplementary file 1 [file Data_Sheet_1.docx]

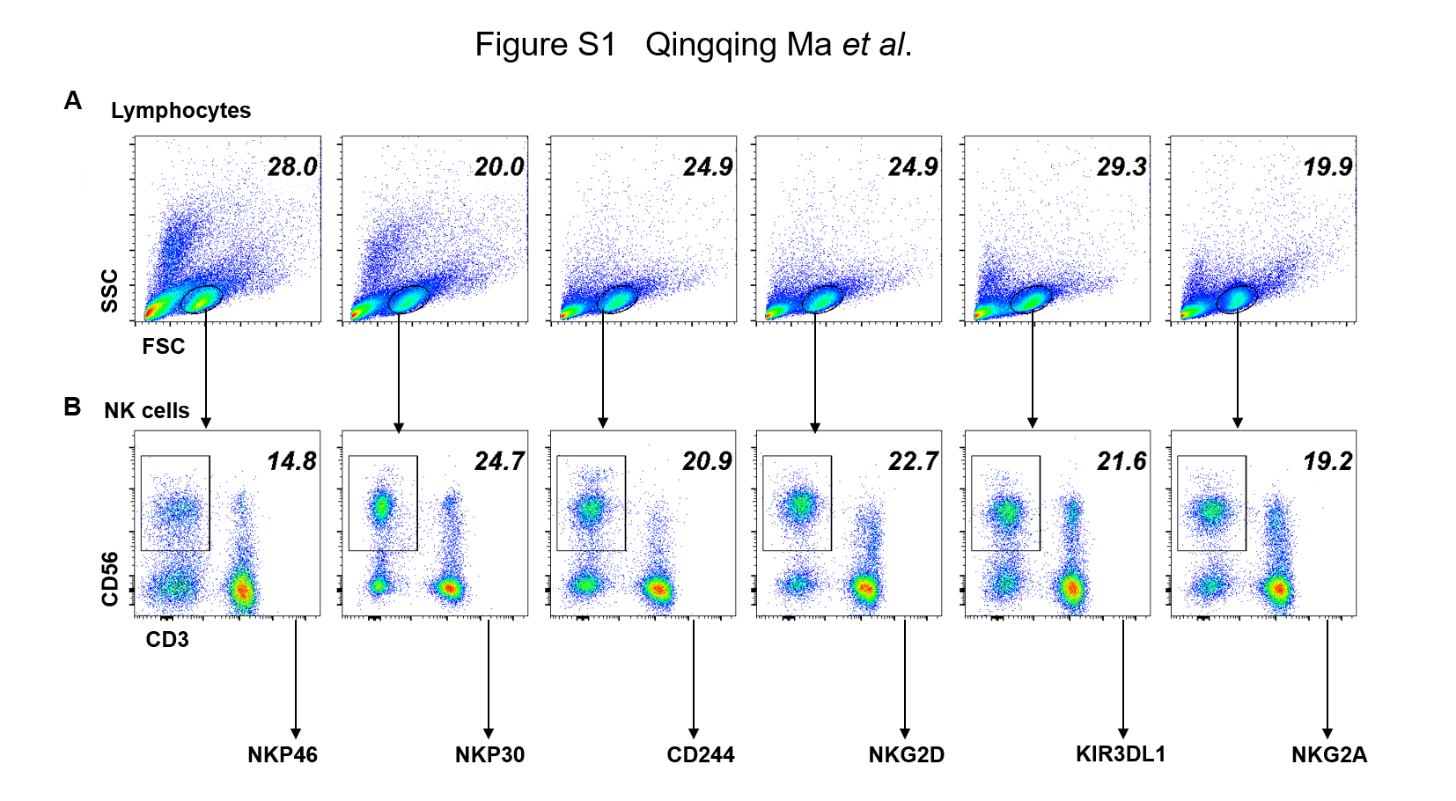


**Fig. S1. Gating information in Figure 1A**

(A)Gating information of lymphocytes in Figure 1A. (B) Gating information of lymphocytes in Figure 1A.


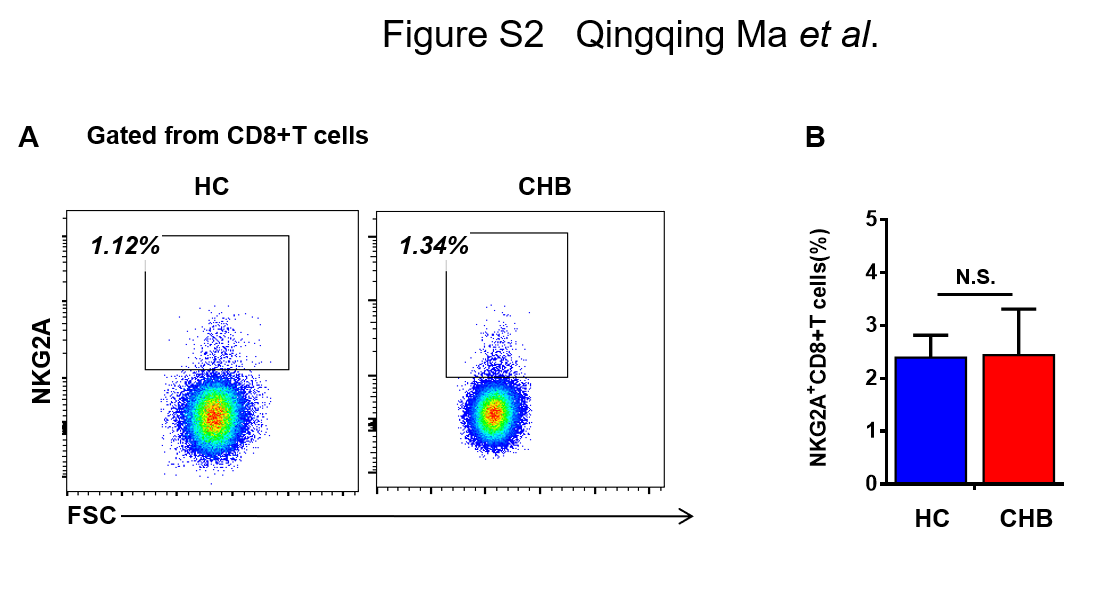


Figure. S2. Expression of NKG2A on CD8+ T cells. (A) Representative flow cytometry plots showing NKG2A expression on CD8+ T cells from patients with CHB and healthy controls. (B) Comparison of the percentage of NKG2A+CD8+ T cells in healthy controls and patients with CHB. Data are representative of more than three independent experiments. Results are presented as the mean ± SEM (n ≥ 3 per group) and an unpaired two-tailed Student’s t-test was conducted; N.S., not significant.


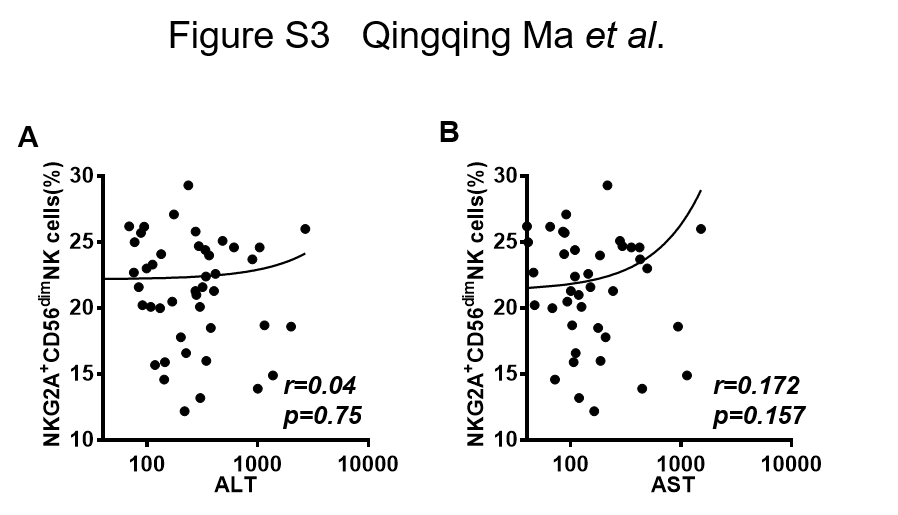


Fig.S3. Correlation between the percentage of NKG2A+CD56dim NK cells and serum transaminases levels in patients with CHB.

(A) Correlation between the percentage of NKG2A+CD56dim NK cells and serum ALT levels in patients with CHB. (B) Correlation between the percentage of NKG2A+CD56dim NK cells and serum AST levels in patients with CHB.


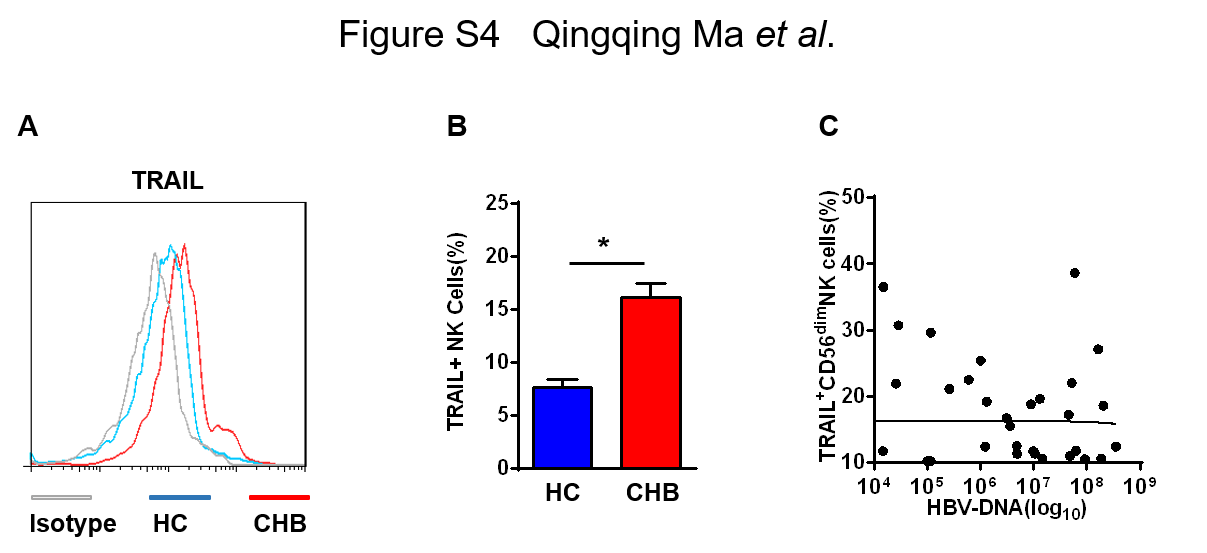


Figure. S4. TRAIL expression patterns on NK cells. (A) Representative flow cytometry plots showing expression of TRAIL and isotype control on NK cells in patients with CHB and healthy controls. (B) Comparison of percentages of TRAIL on NK cells in healthy controls and patients with CHB patients. (C) Correlation between the percentage of TRAIL+CD56dim NK cells and serum HBV-DNA levels in patients with CHB. Data are representative of more than three independent experiments. Results are presented as the mean ± SEM (n ≥ 3 per group) and unpaired two-tailed Student’s t-tests or Spearman’s correlation coefficients were conducted; *p < 0.05.


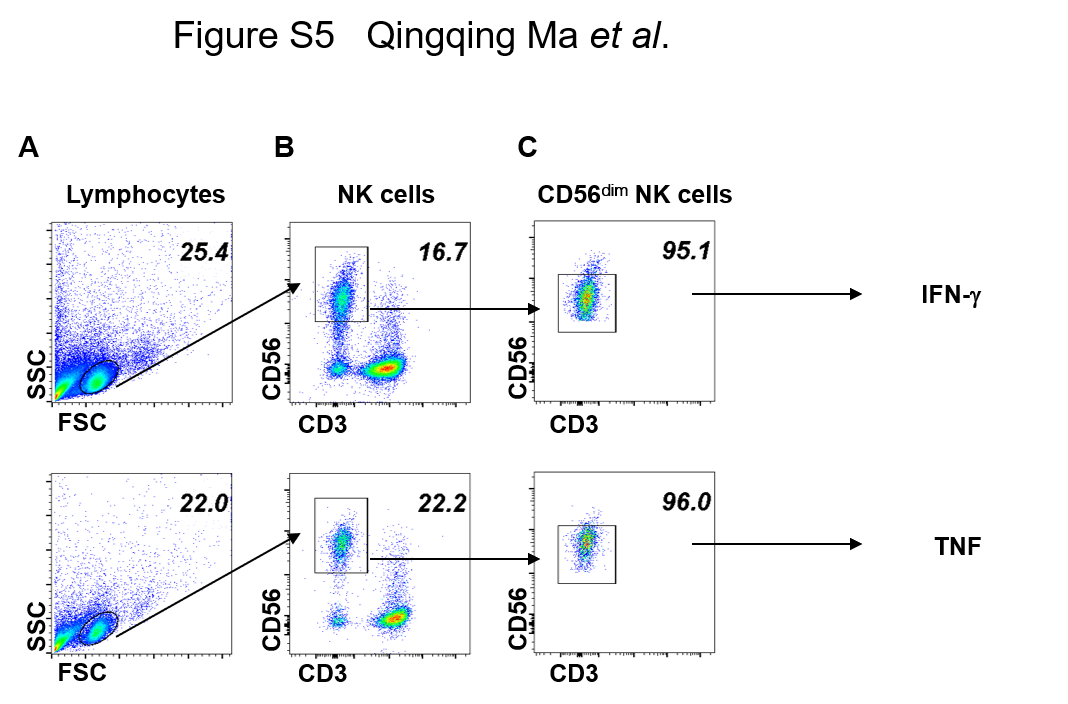


Fig. S5. Gating information in Figure 3A

(A)Gating information of lymphocytes in Figure 3A . (B) Gating information of NK cells in Figure 3A . (C) Gating information of CD56dimNK cells in Figure 3A .


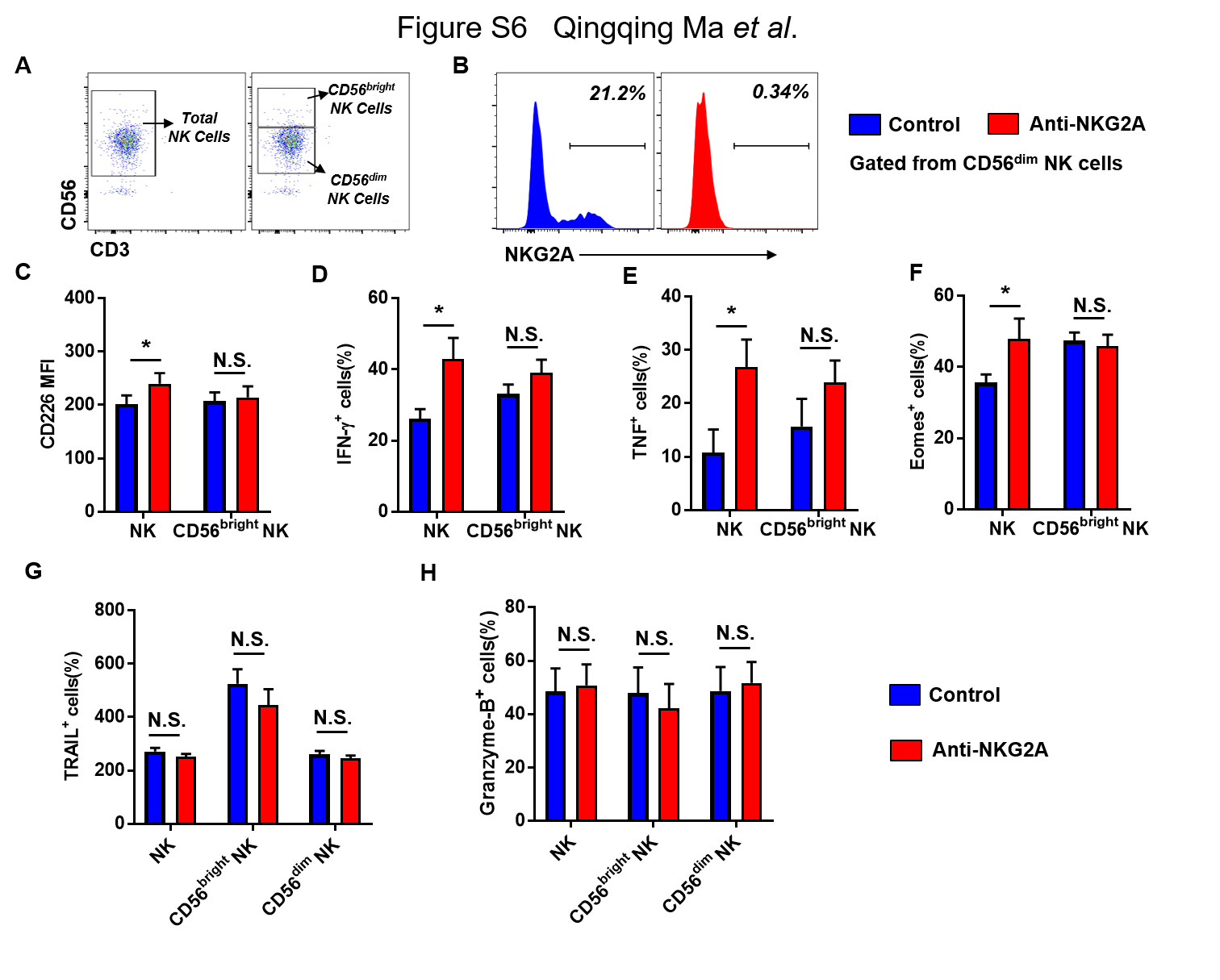


Fig. S6. TRAIL and Granzyme-B expressed on CD56dim NK cells after blockade of NKG2A signaling in patients with CHB.

(A-F) NK cells isolated from patients with CHB were cultured in DMEM supplemented with 10% FBS and 100 IU/ml IL-2, with anti-human NKG2A antibody or control IgG. After 3 days, the phenotype and function of NK cells were analyzed by flow cytometry. (A) Sequential strategy for gating CD56dim , CD56bright and total NK cells from lymphocytes with monitoring via flow cytometry. (B) Representative plots of NKG2A expressed in CD56dim NK cells after NK cells were cultured with anti-human NKG2A antibody or control IgG. (C) Expression of CD226 in CD56bright NK cells after isolated CHB NK cells were cultured with anti-human NKG2A antibody or control IgG. (D) Expression of IFN-g in total NK and CD56bright NK cells after isolated CHB NK cells were cultured with anti-human NKG2A antibody or control IgG. (E) Expression of TNF in total NK and CD56bright NK cells after isolated CHB NK cells were cultured with anti-human NKG2A antibody or control IgG. (F) Expression of Eomes in total NK and CD56bright NK cells after isolated CHB NK cells were cultured with anti-human NKG2A antibody or control IgG. (G) Expression of TRAIL on total NK cells, CD56bright and CD56dim NK cells after isolated CHB NK cells were cultured with anti-human NKG2A antibody or control IgG. (H) Expression of granzyme-B on total NK cells, CD56bright and CD56dim NK cells after isolated CHB NK cells were cultured with anti-human NKG2A antibody or control IgG. Results are presented as the mean ± SEM (n ≥ 3 per group) and unpaired/paired two-tailed Student’s t-tests were conducted. *p < 0.05, N.S., not significant.


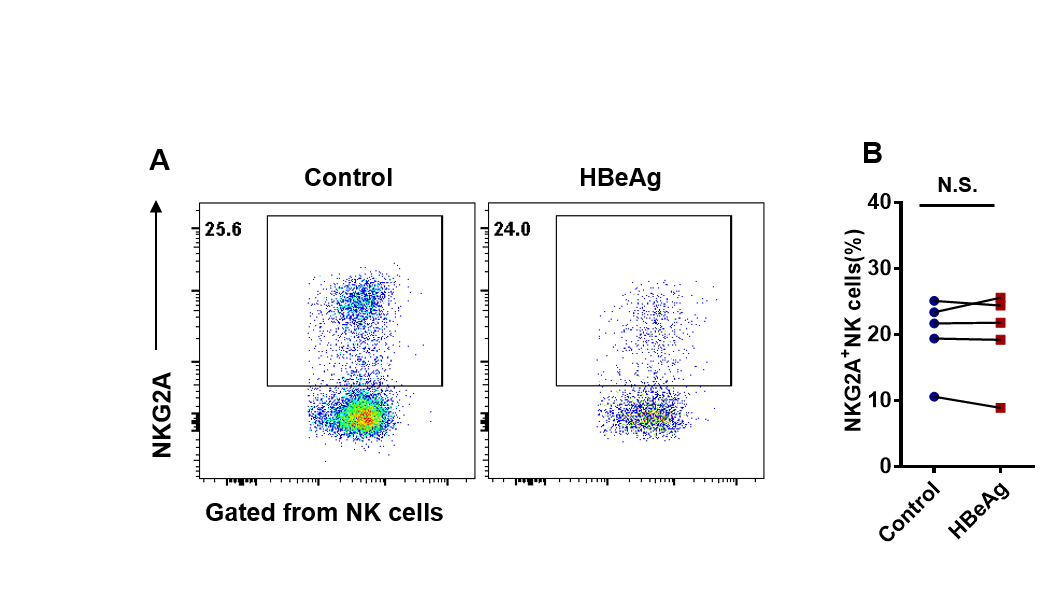


Fig. S7. The expression of NKG2A on NK cells after directly stimulated by HBeAg in vitro

(A-B)1 × 105 NK cells were purified from healthy donors and then cultured in the presence of 10% FBS with or without 500 ng/ml HBeAg for 3 days.(A) Representative flow cytometry plots showing expression of NKG2A on NK cells. (B) Comparison of the percentages of NKG2A on NK cells with or without directly stimulated by HBeAg, N.S., not significant.
